# Time’s imprint on the left atrium: aging and atrial myopathy

**DOI:** 10.20517/jca.2024.23

**Published:** 2025-03-20

**Authors:** Dylan J. Gyberg, Ravi B. Patel, Michael J. Zhang

**Affiliations:** 1 Cardiovascular Division, Department of Medicine, University of Minnesota Medical School, Minneapolis, MN 55455, USA.; 2 Lillehei Heart Institute, University of Minnesota Medical School, Minneapolis, MN 55455, USA.; 3 Division of Cardiology, Department of Medicine, Northwestern University Feinberg School of Medicine, Chicago, IL 60611, USA.

**Keywords:** Atrial fibrillation, atrial myopathy, left atrial reservoir strain, senescence

## Abstract

Aging is a primary driver of atrial remodeling and dysfunction, and contributes to the increasing prevalence of atrial myopathy in the aging population. Atrial myopathy, characterized by structural, functional, and electrophysiological abnormalities of the atria, is a key pathological process underlying adverse cardiovascular outcomes such as atrial fibrillation (AF), heart failure with preserved ejection fraction (HFpEF), and ischemic stroke. Although these outcomes are often treated as distinct clinical entities, emerging evidence suggests that they may represent symptomatic manifestations of an underlying atrial disease process. Aging promotes atrial myopathy through multiple mechanisms, including inflammation, extracellular matrix remodeling, electrophysiological alterations, cellular senescence, epigenetic modifications, and non-coding RNA regulation. These changes collectively lead to atrial fibrosis, impaired mechanical function, conduction abnormalities, and a prothrombotic state. Despite its clinical significance, atrial myopathy remains an underrecognized entity, with current management strategies primarily focusing on treating its downstream complications rather than the underlying disease. Advances in imaging techniques, biomarker discovery, and molecular research have the potential to improve the early detection and risk stratification of atrial myopathy, paving the way for novel therapeutic strategies. In this review, we discuss the structural, mechanical, electrophysiological, and metabolic changes that occur in the aging atrium, explore the cellular and molecular mechanisms that drive these changes, and highlight recent advances in diagnostic and therapeutic approaches. By shifting the focus from managing AF and HFpEF to targeting the underlying atrial myopathy, we can unlock new avenues for prevention and treatment, ultimately improving cardiovascular health in the aging population.

## Graphical Abstract

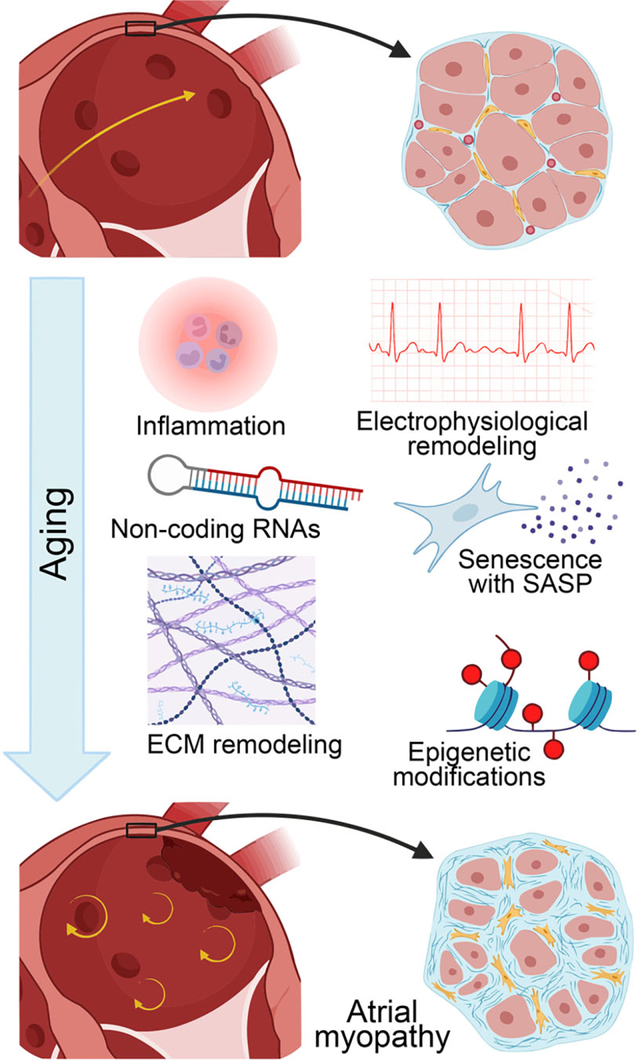

## INTRODUCTION

Populations worldwide are aging at a rapid rate. By 2030, one in six people globally will be aged 60 years or older, marking a substantial increase in the aging population^[1]^. As the global population continues to age, the prevalence of cardiovascular diseases such as atrial fibrillation (AF) and heart failure with preserved ejection fraction (HFpEF) has increased^[2,3]^. These conditions often coexist and are linked by atrial myopathy, a clinical entity defined as structural, functional, and electrophysiological changes in the atria that may underlie adverse cardiovascular outcomes such as AF, HFpEF, and ischemic stroke^[4–8]^.

Aging drives significant left atrial changes, including enlargement, fibrosis, impaired mechanical function, and conduction abnormalities. The clinical implications of atrial myopathy are profound; it is associated with an increased risk of heart failure, AF, stroke, and dementia^[9–12]^. Despite its clinical importance, current therapeutic strategies often only target the clinical sequelae rather than the underlying atrial myopathy, leaving many with subclinical disease untreated. Addressing atrial myopathy itself could enable earlier intervention and improved outcomes.

This review explores the impact of aging on the left atrium, detailing structural, mechanical, electrophysiological, histological, and metabolic changes, as well as the underlying mechanisms driving these changes. We also examine clinical manifestations and challenges in identifying and managing atrial myopathy in older adults. By emphasizing early detection and intervention, we aim to highlight potential research and therapeutic opportunities.

## HEALTHY *VS*. UNHEALTHY ATRIAL AGING

Aging is an inevitable biological process, but its trajectory varies significantly among individuals. Healthy aging refers to the preservation of physiological function and the absence of disease-related impairments despite advancing age. In the cardiovascular system, this concept extends to healthy atrial aging, where the atria maintain their structural integrity, mechanical function, and electrophysiological stability over time. In contrast, unhealthy atrial aging is characterized by increased fibrosis, impaired mechanical function, elevated arrhythmogenic potential, and a prothrombotic state, predisposing individuals to conditions such as AF, HFpEF, and stroke^[6–8]^. Understanding the factors that differentiate these aging trajectories is critical for identifying modifiable risk factors and developing preventative strategies.

A key distinction in aging research is the difference between chronological aging and biological aging. Chronological aging simply reflects the passage of time, whereas biological aging refers to the cumulative impact of genetic, environmental, and lifestyle factors on physiological function. In the context of atrial aging, individuals of the same chronological age may exhibit vastly different levels of atrial health due to differences in biological aging.

Several risk factors contribute to accelerated atrial aging, leading to an increased burden of myopathic changes and its associated complications. Diabetes mellitus promotes atrial fibrosis, impaired relaxation, conduction abnormalities, and increased vulnerability to atrial arrhythmias^[13,14]^. These changes are mediated at least in part by advanced glycation end products and their associated receptor and upregulation of connective tissue growth factor^[15]^. Cigarette smoking is another major contributor to accelerated atrial aging; nicotine-induced upregulation of collagen III promotes atrial fibrosis at a younger age, and there is a strong correlation between pack-years and the extent of atrial fibrosis^[16]^. Hypertension, a common comorbidity in aging adults, is associated with impaired left atrial function as measured by speckle-tracking echocardiography, even after adjustment for left ventricular size and function^[17]^. In addition to environmental and metabolic risk factors, genetic predisposition also plays a role in determining atrial aging trajectories. Variants in genes such as natriuretic peptide precursor A, myosin light-chain 4, and titin have been implicated in pathological atrial remodeling^[18–20]^.

Another notable contributor to unhealthy atrial aging is cardiac amyloidosis, which is characterized by the extracellular deposition of misfolded amyloid proteins in the myocardium. This typically occurs as either light chain amyloidosis or transthyretin amyloidosis. Atrial amyloidosis impairs atrial function and increases the risk of AF and thromboembolism^[21]^. Notably, LA mechanical function is severely impaired in cardiac amyloidosis^[22]^. An analysis of explanted hearts from five patients with transthyretin cardiac amyloidosis revealed mild-moderate subendocardial fibrosis in all samples^[23]^. AF is frequently observed in cardiac amyloidosis, with one study reporting a prevalence of 44% among 238 patients^[24]^. Patients with atrial amyloidosis also have a high incidence of intracardiac thrombosis, even in the absence of AF or despite adequate anticoagulation, placing them at significant risk for thromboembolic events^[25–28]^. A retrospective study of 406 patients with cardiac amyloidosis found that 7.6% experienced an arterial thromboembolic event, with one-third of these cases occurring in individuals who were in sinus rhythm and had no history of AF^[29]^.

## EFFECTS OF AGING ON THE ATRIA

We start by reviewing the effects of aging on atrial structure, mechanical function, electrophysiological function, and metabolic function [Table 1].

### Structure

Aging leads to significant structural and histological changes in the atria, which contribute to the development of atrial myopathy in older individuals. Morphologically, the size of the left atrium increases with age, a phenomenon attributed to both dilation and hypertrophy of the atrial wall^[30–32]^. This enlargement is a compensatory response to increased atrial pressure and volume overload, commonly due to age-related conditions such as uncontrolled hypertension and left ventricular diastolic dysfunction^[33,34]^. Histologically, one of the most prominent age-related changes is the increased fibrosis within the atrial tissue, contributing to decreased atrial compliance and arrhythmogenic substrate^[35–37]^. Concurrently, there is a decrease in myocyte density and an increase in myocyte hypertrophy, which likely contributes to the impaired atrial mechanical function commonly observed in older individuals^[38–40]^. Furthermore, inflammatory cell infiltration is observed more frequently, contributing to pathological atrial remodeling^[41,42]^. The right atrium also undergoes age-related remodeling, including enlargement and fibrosis^[43,44]^. However, this review primarily focuses on left atrial aging because the predominant relevant clinical outcomes - AF, HF, and stroke - are primarily associated with left atrial myopathy.

### Mechanical function

The left atrium is a major contributor to left ventricular filling. It serves as a reservoir for pulmonary venous return during ventricular systole, a conduit during early ventricular diastole, and a booster pump that augments late ventricular filling during ventricular diastole^[45]^. Aging is associated with a decrease in left atrial reservoir strain, indicative of impaired left atrial compliance^[30,46–50]^. This decline in reservoir function is likely, in part, due to atrial fibrosis, which has been shown to increase with age^[35–37]^. Conduit function, much like reservoir function, also seems to decrease significantly with increasing age^[30,46–48,50]^. Conversely, there often appears to be a compensatory increase in left atrial booster function with advancing age, suggesting that active ventricular filling plays a larger role in maintaining preload^[30,50,51]^. Collectively, these findings reflect alterations in left atrial mechanical function with increasing age [Table 2].

### Electrophysiological function

Age-related electrophysiological changes in the atria are clinically significant and are often reflected in distinct alterations in electrocardiogram (ECG) patterns and electrical mapping studies. P wave parameters, in particular, can offer insight into atrial structure, size, and electrical activation^[52]^. Abnormal P-wave terminal force in V1, characterized by a significant negative component of the P wave in V1, is more frequently observed with increasing age^[53,54]^. This abnormal P wave morphology is thought to reflect left atrial enlargement or, as more recent research suggests, disruption of posterior interatrial conduction pathways due to structural remodeling and atrial fibrosis^[52]^. Additionally, in older individuals, the P-wave duration on ECG, which represents atrial depolarization, is often prolonged, indicating delayed intra-atrial conduction^[44,55]^. This is accompanied by an increase in P-wave dispersion, a marker of heterogeneous atrial conduction^[49]^. Abnormal P-wave terminal force, as well as increases in both P-wave duration and dispersion, have been associated with a higher risk of AF^[53,54,56–59]^. Electrical mapping of the atria in older adults frequently reveals areas of low voltage and conduction block, consistent with atrial fibrosis and remodeling^[60,61]^. These areas can be considered markers of arrhythmogenic tissue, contributing to the development and maintenance of atrial arrhythmias^[62–64]^.

### Metabolism

Age-related metabolic changes in the atria are characterized by a decline in mitochondrial function and disruptions in energy transfer pathways, contributing to atrial vulnerability. A recent study demonstrated significant downregulation of genes coding for mitochondrial proteins in the atria, particularly those involved in oxidative phosphorylation^[65]^. This downregulation was most pronounced in complex I subunits, resulting in reduced complex I activity and a decline in the respiratory capacity of mitochondria oxidizing NADH-dependent substrates. Additionally, using advanced ^18^O stable isotope-based phosphometabolomic technology, another study revealed impaired phosphotransfer through adenylate kinase, creatine kinase, glycolytic pathways, and the glycerol-3-phosphate shuttle in the atrial myocardium of aged rats, indicating impaired energetic communication and ATP cycling^[66]^. This energetic deficit may increase the susceptibility of aged atria to stress. Further supporting the role of metabolic alteration in atrial aging, treatment with empagliflozin in aged spontaneously hypertensive rats was found to reduce left atrial dilation and attenuate atrial fibrosis^[67]^. Interestingly, empagliflozin also significantly decreased the age-related upregulation of peroxisome proliferator-activated receptor alpha and acyl-CoA dehydrogenase medium chain, both of which are involved in fatty acid metabolism, suggesting a potential therapeutic role in modulating metabolic pathways to mitigate age-related atrial remodeling. These findings underscore the critical impact of metabolic dysfunction on the aging atrium and its potential as a therapeutic target.

In summary, aging leads to significant changes in the atria that contribute to the development of atrial myopathy. Structural enlargement, mechanical dysfunction, electrophysiologic alterations, and metabolic dysfunction are common in advanced age. These changes are underpinned by histological modifications which compromise atrial function. Collectively, these factors illustrate how aging predisposes the atria to pathological remodeling, ultimately increasing the risk of atrial myopathy.

## MECHANISMS LINKING AGING WITH ATRIAL MYOPATHY

**Figure 1.**
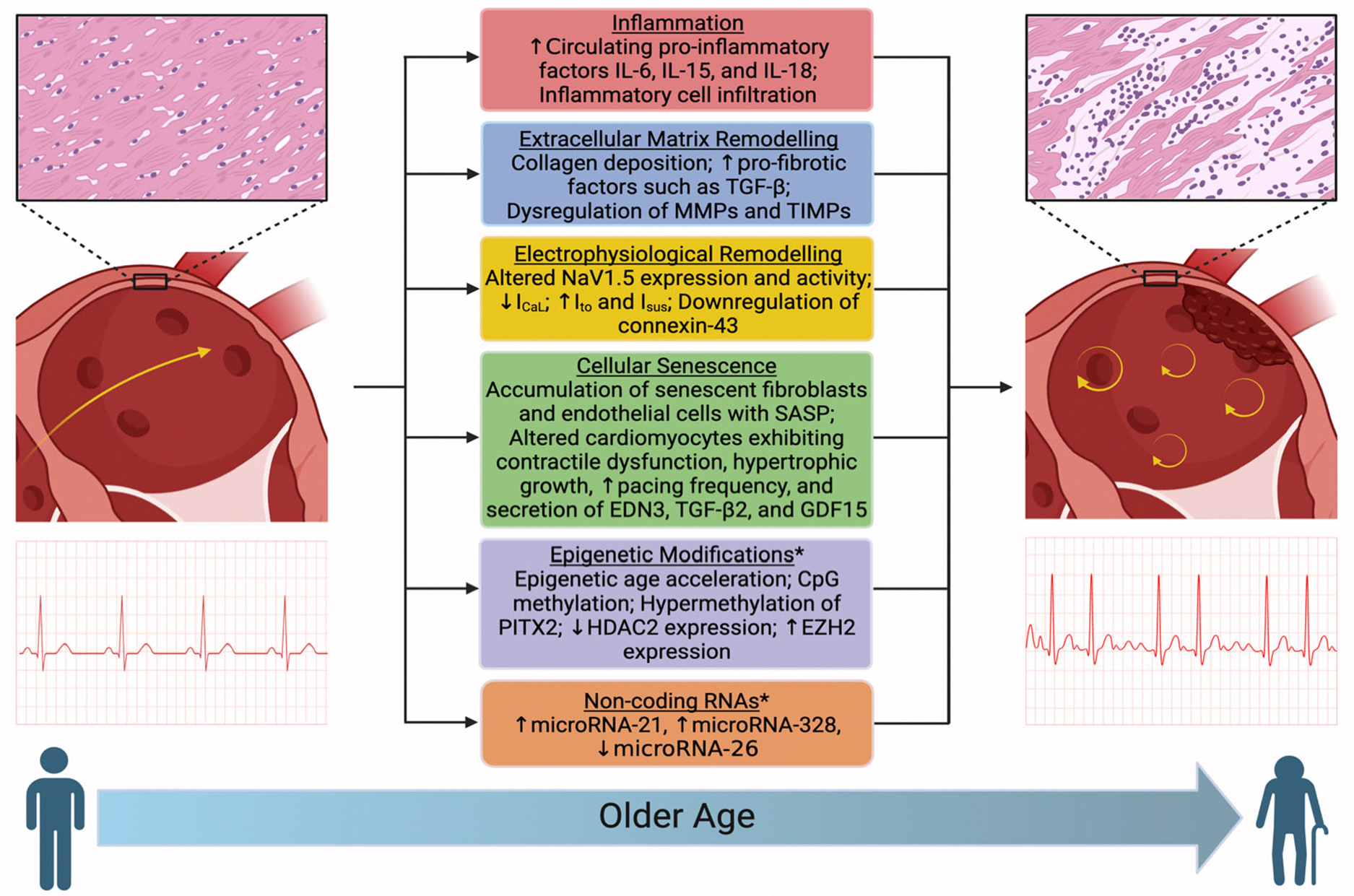
Mechanisms contributing to age-related atrial remodeling. Several mechanisms contribute to age-related pathological atrial remodeling: (1) a chronic inflammatory state marked by elevated circulating pro-inflammatory cytokines, including interleukin (IL)-6, -15, and -18; (2) an increase in circulating pro-fibrotic factors, such as transforming growth factor (TGF)-beta, along with dysregulation of matrix metalloproteinases (MMPs) and tissue inhibitors of metalloproteinases (TIMPs); (3) electrophysiological changes, including altered expression and activity of NaV1.5, decreased L-type calcium current (I_CaL_), increased transient outward (I_to_) and sustained (I_sus_) potassium currents, and downregulation of connexin-43; (4) accumulation of senescent fibroblasts and endothelial cells with a senescence-associated secretory phenotype (SASP), and altered cardiomyocytes that exhibit contractile dysfunction, hypertrophic growth, increased pacing frequency, and secretion of endothelin-3 (EDN3), TGF-beta 2, and growth differentiation factor 15 (GDF15); (5) epigenetic modifications, such as CpG site methylation changes, hypermethylation of paired-like homeodomain 2 (PITX2), and altered expression of histone-modifying enzymes histone deacetylase 2 (HDAC2) and enhancer of zeste homolog 2 (EZH2); and (6) dysregulation of non-coding RNAs, including upregulation of microRNA-21 and microRNA-328, and downregulation of microRNA-26. Collectively, these mechanisms lead to the replacement of healthy myocardium with fibrotic tissue, the development of inflammatory infiltrates, the formation of arrhythmogenic foci, and a prothrombotic state. *These modifications are more specific to atrial fibrillation than aging. Further investigation is needed in this area. Created in BioRender. Gyberg, D. (2005) https://BioRender.com/y38x200.

Age-related inflammation, extracellular matrix remodeling, electrophysiological remodeling, cellular senescence, epigenetic modifications, and non-coding RNAs contribute to pathological atrial remodeling [Figure 1]. Collectively, these mechanisms of age-related atrial myopathy alter the structural integrity of the atrial myocardium and disrupt the normal function of atrial cardiomyocytes, fibroblasts, and endothelial cells, thus leading to pathological atrial remodeling.

### Inflammation

Inflammation plays a pivotal role in the mechanisms that link aging to atrial myopathy, contributing significantly to the structural and functional deterioration of the atrial myocardium. As the body ages, there is a chronic low-grade inflammatory state, often referred to as “inflammaging”, characterized by increased levels of circulating pro-inflammatory factors such as interleukin-6, interleukin-15, and interleukin-18^[68]^. This persistent inflammatory state promotes the recruitment of immune cells to atrial tissue^[41,42]^. The presence of these immune cells in the atria exacerbates the production of fibrotic factors, enhancing collagen deposition and the formation of fibrotic patches within the atrial myocardium^[69–71]^. Such fibrotic remodeling impairs atrial compliance and contractility, and disrupts the normal electrical conduction pathways, increasing the risk for arrhythmias such as AF^[72,73]^. These processes underline the critical role of inflammation as a driver of pathological remodeling associated with atrial myopathy in aging adults.

### Extracellular matrix remodeling

Extracellular matrix remodeling is a central mechanism in the progression of atrial myopathy in the aging heart, marked by the excessive accumulation of collagen within atrial tissue^[35–37]^. As individuals age, the regulatory balance between matrix synthesis and degradation becomes disrupted, often tipping in favor of matrix deposition. This shift is mediated by an increase in pro-fibrotic factors such as transforming growth factor beta and dysregulation of matrix metalloproteinases and tissue inhibitors of matrix metalloproteinases^[74,75]^. These fibrotic areas contribute to atrial mechanical dysfunction, particularly impaired reservoir function^[73]^. Additionally, the fibrotic remodeling of the atrial myocardium impairs normal atrial electrical impulse conduction and promotes electrical heterogeneity, thereby increasing the risk for atrial arrhythmias^[72,76]^.

### Electrophysiological remodeling

Beyond the conduction disturbances associated with fibrosis, age-related electrophysiological remodeling in the atria is driven by a complex interplay of molecular and cellular changes that alter the electrical properties of atrial cardiomyocytes. Research on the effects of aging on the expression and function of ion channels in the atria is limited and sometimes contradictory. For instance, studies indicate that NaV1.5, the predominant cardiac voltage-gated sodium channel, is upregulated in the atria of aged rats^[77]^. Conversely, research in aged canines shows no change in sodium current density or structural remodeling of NaV1.5 in aged atrial cells compared to young controls^[78]^. However, the same study did note a significant acceleration into the inactivated state and an enhanced use-dependent decrease in peak current in aged right atrial cells, but interestingly not in left atrial cells. Additionally, findings in aged canines revealed that right atrial cells exhibit reduced L-type calcium currents and augmented transient outward and sustained potassium currents^[79]^. Although the body of literature is limited, these studies collectively suggest that aging is associated with alterations in ion channel expression and function in the atria, which may contribute to increased arrhythmogenic potential. Furthermore, there is evidence that aging is associated with the downregulation of connexin-43, a gap junction protein, in the atria^[80]^. This likely results in impaired electrical conduction between atrial myocytes. These age-related modifications in ion channel dynamics and gap junction connectivity collectively dimmish the atrial myocardium’s electrical stability.

### Cellular senescence

Cellular senescence is characterized by a state of growth arrest, resistance to apoptosis, and the acquisition of a senesce-associated secretory phenotype, which includes the secretion of pro-inflammatory molecules and enzymes involved in extracellular matrix remodeling^[81]^. Senescent cells are distinct from quiescent cells, which are temporarily non-dividing but can re-enter the cell cycle, and from terminally differentiated cells, which are permanently non-dividing but reach this state through a developmentally programmed process^[82]^. Senescent cells accumulate in the heart over time and contribute to age-related cardiovascular diseases^[83]^. The impact of cellular senescence on the cardiovascular system is complex; however, recent evidence suggests it may adversely affect atrial function. A study by Jesel *et al.* investigated right atrial appendage tissue from patients with sinus rhythm, paroxysmal AF, or permanent AF^[84]^. They observed a progressive increase in the expression of senescence markers p16 and p53 across these groups, indicating an association between AF progression and human atrial senescence burden^[84]^. The downstream effects of cellular senescence depend on the cell type. Cardiomyocytes, being terminally differentiated cells, do not undergo senescence in the conventional sense. Instead, cardiomyocyte senescence is characterized by contractile dysfunction, hypertrophic growth, increased pacing frequency, mitochondrial dysfunction, and DNA damage^[85]^. Although senescent cardiomyocytes do not exhibit a typical senescence-associated secretory phenotype, they release factors such as endothelin-3, transforming growth factor beta-2, and growth differentiation factor 15 that promote myofibroblast activation and cardiomyocyte hypertrophy^[86]^. Senescent atrial fibroblasts likely contribute to cardiac remodeling by expressing increased levels of fibrotic factors such as collagen, transforming growth factor beta, and matrix metalloproteinase-2 and 9^[87]^. Additionally, senescent atrial endothelial cells show enhanced expression of vascular cell adhesion molecule-1, transforming growth factor beta, matrix metalloproteinase-2 and -9, and tissue factor, potentially contributing to atrial inflammation, remodeling, and a prothrombotic state^[88]^. Collectively, these effects likely contribute to aging-associated atrial myopathy.

### Epigenetic modifications

Aging is characterized by several epigenetic changes, including loss of histones, an imbalance between activating and repressive histone modifications, alterations in DNA methylation, and chromatin remodeling^[89]^. Growing evidence suggests that these epigenetic changes play a significant role in age-related cardiovascular diseases^[90]^. A recent study evaluated the association between epigenetic age acceleration - a measure of biological aging based on epigenetic modifications - and the risk of incident AF^[91]^. The study found that increases in epigenetic age acceleration were significantly associated with a higher risk of developing AF, even after adjusting for chronological age and other risk factors. These findings suggest that age-related epigenetic modifications may contribute to the pathogenesis of AF, highlighting a potential mechanism linking biological aging to atrial myopathy. While specific age-related epigenetic modifications that directly link aging to atrial myopathy have yet to be fully identified, existing evidence connects certain epigenetic alterations with AF and atrial remodeling. For instance, a methylome-wide association study in a community-based cohort identified seven CpG sites that were associated with AF, indicating that DNA methylation may contribute to AF arrhythmogenesis^[92]^. Additionally, hypermethylation of paired-like homeodomain 2, a key regulatory gene, in the left atrium has been associated with AF and LA dilation^[93]^. Beyond changes in DNA methylation, histone modifications also appear to contribute to the pathogenesis of atrial myopathy. Downregulation of histone deacetylase 2 is associated with AF and has been shown to cause altered potassium channel expression and action potential prolongation^[94]^. Moreover, the expression of enhancer of zeste homolog 2, a histone-lysine N-methyltransferase enzyme, is upregulated in the atrial tissue of patients with atrial fibrillation, and this increase is associated with significant atrial fibrosis and fibroblast differentiation^[95]^. Similar upregulation of enhancer of zeste homolog 2 and increased methyltransferase activity were observed in a mouse model of atrial fibrosis induced by angiotensin II infusion. Together, these findings suggest that epigenetic modifications, which are hallmarks of aging, likely contribute to age-related atrial remodeling and the development of atrial myopathy.

### Non-coding RNAs

Non-coding RNAs, including microRNAs and long non-coding RNAs, have recently described roles in age-related cardiovascular disease^[96,97]^. In mice, microRNA-22 is upregulated with aging and promotes cellular senescence and fibroblast activation^[98]^. Similarly, mircoRNA-34a, which is also upregulated with age, contributes to age-related cardiac cell death and functional decline by inhibiting PNUTS, a regulator that protects against telomere shortening^[99]^. Conversely, SARRAH, a long non-coding RNA that supports cardiomyocyte survival by forming a triple helix structure with promoter regions to enhance the expression of cardiac survival genes, is downregulated with aging^[100]^. Although research on aging-regulated non-coding RNAs in atrial myopathy remains limited, several non-coding RNAs have been implicated in pathological atrial remodeling. MicroRNA-21, which is upregulated in the left atrium of AF patients and positively correlated with atrial collagen content, plays a role in angiotensin II-mediated atrial remodeling^[101]^. MicroRNA-328, another microRNA elevated in AF, contributes to adverse electrical remodeling by targeting L-type calcium channel genes^[102]^. Additionally, microRNA-26 has been identified as a determinant of AF vulnerability, as it regulates the expression of KCNJ2, a key component of inward rectifier potassium current^[103]^. Further research is needed to identify non-coding RNAs involved in age-related atrial myopathy.

## CLINICAL IDENTIFICATION AND MANIFESTATIONS OF ATRIAL MYOPATHY IN THE CONTEXT OF AGING

Identifying atrial myopathy in the aging population requires a comprehensive approach, integrating clinical assessment, imaging, and electrophysiological studies. A thorough history and physical examination focusing on symptoms of arrhythmias, HF, and thromboembolic events is essential. Identifying risk factors such as hypertension, valve disease, diabetes, and previous cardiac events is critical, as these conditions often coexist with atrial myopathy in aging adults^[104]^. Electrocardiography remains the cornerstone for detecting AF and other supraventricular tachycardias. Abnormal P-wave terminal negative force in V1 may reflect left atrial enlargement of disruption of interatrial conduction pathways due to fibrotic remodeling. Furthermore, invasive electrophysiological studies such as atrial voltage mapping can identify areas of low-voltage reflecting atrial fibrosis and arrhythmogenic foci^[62,64]^.

Historically, electrocardiography was the primary method for identifying atrial myopathy. However, echocardiography has emerged as a more comprehensive tool for evaluating left atrial structure and function. Transthoracic echocardiography enables the measurement of atrial volume and strain, with enlarged atria and reduced strain being hallmarks of myopathy^[105–108]^. Left atrial strain, especially reservoir strain, has gained prominence as a reliable marker of atrial function^[109–112]^. Although LA strain measurement is not yet widely implemented in clinical practice, the standardization of deformation imaging techniques and ongoing refinement of normal reference ranges are paving the way for its future widespread adoption^[30,46,47,113–115]^. However, despite demonstrating statistically significant changes in mean values across studies, left atrial strain often exhibits substantial overlap in standard deviations, complicating the interpretation of individual patient measurements. Further validation is needed to enhance its utility in routine clinical assessment. Doppler studies provide insights into diastolic function and atrial contribution to ventricular filling. Pulsed wave Doppler measurements of peak transmitral flow velocity during late diastolic filling have been used in several studies to assess left atrial contractile function^[116,117]^. However, the utility of this measure is limited, as transmitral flow patterns are influenced by various factors, including age, heart rate, loading conditions, and left ventricular diastolic properties^[118]^. Tissue Doppler imaging of the mitral annulus during atrial contraction is another measure of left atrial function, and has been shown to correlate with left atrial ejection fraction, ejection force, and kinetic energy^[119]^. Cardiac magnetic resonance imaging (MRI) offers detailed visualization of atrial structure and fibrosis, with late gadolinium enhancement identifying areas of atrial scarring^[120]^. Computed tomography imaging can evaluate atrial anatomy, wall thickness, and is a reliable method for detecting thrombi within the left atria or left atrial appendage^[121,122]^.

Several serum biomarkers that may indicate atrial myopathy have been identified, though none have been widely adopted specifically for this purpose in clinical practice. In a study of HFpEF patients enrolled in the RELAX trial, elevated levels of NT-proBNP, endothelin-1, and troponin I were found to correlate with reduced left atrial reservoir and contractile strain, suggesting that these markers may reflect underlying atrial dysfunction^[123]^. Furthermore, repeated biomarker measurements at the 24-week follow-up demonstrated that higher left atrial reservoir strain was independently associated with a reduction in NT-proBNP, indicating a potential dynamic relationship between atrial strain and circulating biomarkers. These findings underscore the potential usefulness of serum biomarkers for monitoring left atrial function.

Atrial myopathy presents various clinical manifestations [Table 3] that become particularly nuanced in the older population due to the combined effects of age-related physiological decline and the prevalence of comorbid conditions^[124,125]^. Among these manifestations, AF stands out as the most common and significant^[6,8,126,127]^. Age is recognized as the most critical risk factor for AF, with epidemiological studies indicating that nearly one-third of individuals aged 85 years or older are affected by this arrhythmia^[128,129]^. In addition to AF, other arrhythmias such as atrial flutter may also develop, further complicating the clinical picture of atrial myopathy in older adults^[130,131]^. The clinical implications of AF are profound, including an increased risk of stroke, HF, and mortality^[132–134]^. Given these severe consequences, there is an ongoing need to improve prevention strategies. Shifting the focus from AF to atrial myopathy may uncover new, potentially more effective preventative approaches by targeting the underlying substrate of atrial dysfunction. Additionally, while current guidelines do not recommend routine screening for AF, this shift in focus could eventually result in sufficient evidence to support screening for atrial myopathy.

HF, particularly HFpEF, is another significant clinical manifestation of atrial myopathy in older individuals. Age is also the leading risk factor for the development of HFpEF, making this condition increasingly prevalent in the aging population^[135]^. Atrial myopathy is one of the most important contributors to disease progression in HFpEF because it leads to left atrial dysfunction, which is closely associated with increased pulmonary vascular resistance and right ventricular dysfunction in HF patients^[7,136]^. Indeed, atrial myopathy in HFpEF is characterized by increased atrial stiffness and higher peak atrial pressures, distinct from other forms of HF^[136]^. Furthermore, recent evidence suggests the existence of a unique HFpEF phenotype characterized by disproportionate atrial myopathy with preserved diastolic function, indicating that atrial myopathy may represent a distinct pathophysiological process in HFpEF development rather than merely a consequence of left ventricular dysfunction^[137]^. Additionally, atrial myopathy may contribute to the development of functional mitral regurgitation in patients with HFpEF, which further reduces cardiac output^[138]^. Atrial arrhythmias, particularly AF, may also develop, reducing the atrial contribution to left ventricular filling and thereby worsening cardiac output^[139]^.

Thromboembolism is another critical manifestation of atrial myopathy, particularly in aging adults, where it poses a significant risk for morbidity and mortality. Atrial myopathy and the associated atrial remodeling create a prothrombotic state, which may increase the risk of cardioembolic strokes, independent of the presence of AF^[6,8,140]^. Brambatti *et al*. suggest that this is the most likely explanation for the lack of temporal association between paroxysmal AF and stroke^[141]^. Recent findings by Tan *et al*. provide compelling evidence for this association. In a study involving 180 aging male mice, 20 mice experienced stroke events despite the absence of AF^[42]^. Notably, these mice exhibited left atrial appendage thrombi, enlarged left atria, and significant endocardial remodeling within the left atrium. These findings underscore the need for careful risk stratification and consideration of anticoagulation in older patients with atrial myopathy, even in the absence of AF, to mitigate the risk of stroke.

## RECENT ADVANCEMENTS

### Emerging technologies

Emerging technologies are poised to significantly enhance the detection and monitoring of atrial myopathy. High-resolution imaging modalities, including advanced echocardiography and cardiac MRI, can provide detailed insights into atrial structure and function, enabling the detection of subtle changes that may indicate early atrial myopathy^[142–144]^. Wearable devices such as smartwatches enable real-time tracking of cardiac rhythms and other physiological parameters, thereby facilitating the early identification of atrial dysfunction. Studies have shown that smartwatches can be used to detect various supraventricular arrhythmias, and can identify AF with relatively high sensitivity and specificity^[145,146]^. Moreover, artificial intelligence-based tools are being rapidly developed to enhance the assessment of atrial function, enabling more accurate detection of subtle atrial abnormalities and early identification of disease. These artificial intelligence tools have demonstrated significant potential in analyzing electrocardiographic and imaging data^[147–150]^. Together, these advancements hold the promise of transforming the clinical management of atrial myopathy, enabling earlier intervention, more precise monitoring, and ultimately improving patient outcomes.

While these emerging technologies provide valuable tools for detecting atrial dysfunction, the ability to directly identify the underlying atrial substrate, such as atrial fibrosis, may enable even earlier detection and more targeted therapeutic strategies for atrial myopathy before overt dysfunction occurs. Late gadolinium enhancement MRI has been proposed as a non-invasive technique to detect areas of fibrotic tissue within the atria^[120,151]^. This imaging modality works by leveraging the differential uptake and washout of gadolinium contrast agent between healthy and fibrotic myocardial tissue; fibrotic regions retain gadolinium longer than normal myocardium due to increased extracellular volume, resulting in areas of hyperenhancement on MRI. While quantification of atrial fibrosis by late gadolinium enhancement MRI has demonstrated some clinical utility in predicting arrhythmia recurrence following catheter ablation of AF, it remains an indirect measure of fibrotic burden, may not fully capture the complexity of the fibrotic substrate, and carries technical challenges that may limit broad implementation^[152]^. Furthermore, its clinical application has yielded mixed results; the DECAAF II trial evaluating MRI-guided fibrosis ablation in conjunction with pulmonary vein isolation did not show a significant reduction in atrial arrhythmia recurrence compared to pulmonary vein isolation alone^[153]^. This outcome underscores the need for further refinement of imaging techniques and more targeted interventions to effectively address the underlying fibrotic process in atrial myopathy.

### Clinical utility of left atrial size and strain measurements

Left atrial size and strain measurements are increasingly recognized as valuable clinical tools for predicting adverse cardiovascular outcomes. Increased left atrial size, a well-established marker of atrial myopathy, has been linked to new-onset congestive HF, postoperative AF, and increased mortality in patients with acute myocardial infarction^[154–156]^. In particular, minimal left atrial volumes, as opposed to maximal volumes, are not affected by longitudinal left ventricular systolic motion and have been more strongly associated with adverse clinical outcomes^[157,158]^. More recently, left atrial strain, particularly reservoir strain, has gained attention as a sensitive indicator of both subclinical and overt cardiovascular conditions. Notably, left atrial strain has emerged as a predictor of subclinical AF in older patients at risk of stroke, offering the potential for early detection and intervention^[109]^. Additionally, decreased left atrial strain has been associated with a higher incidence of ischemic stroke, highlighting its potential role in stroke risk stratification^[110]^. Furthermore, in patients with congestive HF, reduced reservoir strain correlates with diminished exercise capacity, reinforcing its utility in assessing functional status^[111]^. Importantly, left atrial reservoir stain has also been identified as a predictor of all-cause mortality and cardiac hospitalization in HF patients^[112]^. These findings underscore the potential of left atrial measurements not only as diagnostic tools but also as prognostic markers that could guide therapeutic strategies and improve patient outcomes in clinical practice.

## CURRENT CHALLENGES

### Lack of diagnostic criteria

Despite the growing recognition of atrial myopathy as a distinct clinical entity, there are currently no established diagnostic criteria to accurately diagnose this condition. This lack of standardized criteria poses a significant challenge in both clinical practice and research, making it difficult to assess the true prevalence, progression, and impact of atrial myopathy. There are well-established markers of atrial myopathy that are identifiable via the use of electrocardiography, cardiac imaging, and electrophysiological studies; however, the absence of a unified diagnostic framework means that these findings are often interpreted in isolation. This gap underscores the need for comprehensive criteria that integrate these markers to facilitate earlier and more accurate identification of atrial myopathy, particularly in older patients where the condition is more prevalent^[128]^. Notably, Kreimer and Gotzmann recently proposed an algorithm for diagnosing atrial myopathy that combines findings from cardiac magnetic resonance imaging, electrophysiological studies, echocardiography, and electrocardiography^[159]^. This algorithm classifies markers of atrial myopathy into three categories: strong evidence (e.g., low-voltage area > 10%), supporting findings (e.g., decreased LA emptying fractions), and uncertain findings (e.g., LA blood flow velocities). This proposed algorithm may provide a potential foundation for developing a robust and reliable approach to diagnosing atrial myopathy.

### Current limitations of LA imaging techniques

Although advancements in imaging techniques have improved the assessment of abnormal LA structure and function, several limitations remain. A review by Olsen *et al.* discusses key challenges in echocardiographic, MRI, and computed tomography assessment of the LA^[160]^. Echocardiographic assessment of LA volumes and strain is hindered by methodological variability, including inconsistencies in chamber delineation, tracking techniques, and ECG reference points. Additionally, LA strain values are influenced by technical factors such as atrial wall structure, pulmonary venous anatomy, the inclusion of the interatrial septum, and vendor-dependent measurement differences. Cardiac magnetic resonance with late gadolinium enhancement imaging provides valuable insights into atrial fibrosis but presents technical challenges. Imaging the thin atrial wall requires precise ECG and respiratory gating, as well as expertise in opitmization of inversion times for myocardial nulling. Post-processing is time-intensive, and the lack of standardized segmentation and thresholding methods limits reproducibility across centers. Similarly, although computed tomography offers detailed structural assessment, its clinical utility in atrial myopathy characterization is limited by radiation exposure and the need for iodinated contrast administration. Collectively, these limitations underscore the need for further methodological standardization to enhance the clinical applicability of these imaging techniques.

### Additional pathophysiologic insights needed

Understanding the intricate pathophysiology of atrial myopathy in the context of aging remains a significant challenge. The interplay between age-related structural, functional, and electrophysiological changes in the atria and broader cardiovascular system is multifaceted, complicating efforts to identify specific molecular targets for intervention and confounding the development of effective therapeutic strategies. Findings from our recent study in which we characterized left atrial function in three different mouse models of atrial myopathy - aging, HFpEF, and angiotensin II infusion - have underscored the complexity of aging-associated atrial myopathy. While all three models demonstrated left atrial mechanical dysfunction, there was a notable discrepancy in atrial fibrillation inducibility: aged mice did not exhibit the increased susceptibility to AF seen in the HFpEF and angiotensin II infusion groups^[48]^. This unexpected finding suggests that the mechanisms linking aging to atrial myopathy may differ fundamentally from those driving other forms of the disease. Further research is needed to elucidate the underlying factors contributing to this discrepancy, potentially involving deeper investigation into age-specific molecular pathways and alterations in atrial tissue composition. Additionally, the aging population is highly heterozygous, with significant variations in comorbidities, genetic predispositions, lifestyle factors, and overall health status. This diversity complicates the generalization of research findings and the development of standardized treatment protocols, necessitating a personalized medicine approach, which is still in its nascent stages.

## FUTURE PERSPECTIVES

### Novel biomarkers

Looking ahead, the identification and validation of biomarkers for atrial myopathy may hold potential for advancing both research and clinical practice. Several biomarkers associated with atrial myopathy have already been identified, but their clinical utility may be limited due to issues of specificity. For example, while elevated levels of troponin I and NT-proBNP have been linked to decreased left atrial reservoir and contractile strain in HFpEF patients, these biomarkers lack specificity because they also indicate other cardiovascular conditions, such as myocardial infarction and HF^[123,161,162]^. Thus, future efforts should focus on discovering more specific biomarkers that can accurately reflect left atrial dysfunction. Despite these challenges, biomarkers may offer a more precise and non-invasive way to identify atrial myopathy, especially in its early stages, when structural and functional changes might not yet be detectable through conventional imaging techniques. With the validation of such biomarkers, clinicians could identify patients at risk of developing atrial myopathy before significant dysfunction occurs. Furthermore, validated biomarkers would be invaluable for stratifying patients for clinical trials and could serve as surrogate endpoints in studies, potentially accelerating the development of new therapeutic agents.

### Therapeutic interventions

Developing effective therapeutic interventions for atrial myopathy poses a significant challenge. Although atrial myopathy is a distinct clinical entity, it often develops secondary to conditions such as hypertension, HFpEF, and mitral valve disease^[17,163,164]^. Early identification and management of these underlying disorders may help slow their progression and mitigate structural and functional changes in the left atrium. However, in the cases when atrial myopathy occurs as a primary disorder, most therapeutic approaches focus on mitigating the downstream consequences of atrial myopathy - such as AF, HF, and thromboembolic events - rather than addressing the underlying atrial pathology.

Several therapies are under investigation for their potential to protect the left atrium from adverse remodeling. For instance, results of the RACE-3 trial indicated that mineralocorticoid receptor antagonists, such as spironolactone, may promote the maintenance of sinus rhythm in patients with AF and HF, suggesting a protective effect on the left atrium^[165,166]^. This effect might be attributed to the antifibrotic properties of spironolactone, which could reduce further pathological atrial remodeling^[167]^. However, it is important to note that spironolactone is not specific to the left atrium and its effects are not limited to this region. Moreover, despite the promising results of the RACE-3 trial, the IMPRESS-AF trial later demonstrated that spironolactone did not improve exercise capacity, E/e’ ratio, or quality of life in patients with chronic AF, but results may have been driven by the fact that AF burden was very high in the trial population^[168]^. Meanwhile, devices aimed at reducing left atrial pressure are also under exploration. For example, the placement of a transcatheter interatrial shunt device has shown promise by reducing pulmonary capillary wedge pressure during exercise in patients with HF and an ejection fraction above 40%^[169]^. Similar interventions, such as a shunt device between the left atrium and coronary sinus, are being developed to alleviate the pressure burden on the left atrium^[170]^. Nonetheless, additional research is necessary to determine whether these devices will lead to improved outcomes for patients with atrial myopathy. Another promising treatment for atrial myopathy targets the underlying causes of impaired atrial function: danicamtiv, a cardiac myosin activator, has been shown to increase ATPase activity and calcium sensitivity in left atrial muscle fibers and improve left atrial function in patients with HF with reduced ejection fraction^[171]^.

Given the accumulation of senescent cells with aging and their contribution to cardiovascular disease, senolytic drugs - which selectively eliminate these cells - hold promise as a therapeutic strategy for mitigating age-related atrial myopathy, though further research is needed to determine their efficacy and safety^[172]^. Finally, several genetic causes of atrial myopathy have been identified, paving the way for future gene-modifying therapies aimed at correcting these underlying genetic defects^[173,174]^.

### Antithrombotic therapy in atrial myopathy

Anticoagulation in patients with atrial myopathy, particularly those with embolic stroke of undetermined source, remains a contentious topic^[175]^. The recent ARCADIA trial evaluated the efficacy of apixaban compared to aspirin for secondary stroke prevention in this population, enrolling 1,015 participants with cryptogenic stroke and markers of atrial myopathy, such as P-wave terminal force on electrocardiography, elevated NT-proBNP levels, or increased left atrial size on echocardiography^[176]^. The trial was halted early for futility after a mean follow-up of 1.8 years, demonstrating no significant difference in recurrent stroke rates between apixaban and aspirin groups (4.4% in both). Several factors might explain the negative outcome of the ARCADIA trial. First, a longer follow-up period with more events might have revealed statistically significant differences between the two groups^[177]^. Second, many participants had hypertension, diabetes, and tobacco use, which increased their baseline risk for atherosclerotic cardiovascular disease, suggesting that recurrent strokes may have been atherosclerotic rather than cardioembolic. Third, the delayed enrollment after the qualifying stroke, along with the mild stroke severity at baseline, may have led to the omission of high-risk patients. Fourth, approximately 46% of participants were enrolled based on NT-proBNP levels alone, a biomarker that may not be sufficiently specific for atrial myopathy^[178]^. Additionally, the possible inclusion of patients with patent foramen ovale, for whom no data were presented, could have influenced the results, because patent foramen ovale-related atrial dysfunction may promote *in situ* thrombosis and not qualify as cryptogenic stroke, potentially affecting the study’s applicability and interpretation^[179]^. Although the ARCADIA trial results were null, it does not mean that we should abandon the idea of anticoagulation in patients with atrial myopathy; instead, the findings provide valuable insights for refining future research in this area.

### Primary prevention of atrial myopathy

Many cardiovascular diseases, such as coronary artery disease, have efficacious preventative therapies in addition to secondary therapeutics. Compared to treating atrial myopathy, preventing the onset of atrial myopathy in the aging population also presents a complex challenge due to the multifactorial nature of the disease. Unlike coronary artery disease, which is driven by atherosclerosis and amenable to targeted interventions like statins or antiplatelet agents, the pathogenesis of atrial myopathy involves diverse mechanisms, including inflammation, oxidative stress, and increased atrial stretch^[8]^. These factors collectively promote atrial remodeling, fibrosis, and electrophysiological changes, all of which are difficult to modify. Thus, while primary prevention of atrial myopathy remains challenging, it underscores the need for more comprehensive and individualized strategies that target the underlying contributors to atrial disease.

## SUMMARY

Aging is a primary driver of atrial myopathy, a condition that underlies AF, HFpEF, and thromboembolism. As the older population continues to expand, understanding the interplay between aging and atrial myopathy is increasingly important. This review has highlighted the structural, mechanical, electrophysiological, histological, and metabolic changes that occur in the left atrium with advancing age, emphasizing their role in the pathogenesis of atrial myopathy. The recognition of atrial myopathy as a distinct clinical entity shifts the focus from solely managing its symptomatic manifestations to addressing the underlying atrial pathology. This paradigm shift has significant implications for both the prevention and treatment of cardiovascular diseases in the aging population. Early detection and intervention strategies targeting subclinical atrial myopathy may help prevent progression to more severe outcomes.

Despite the growing evidence linking aging to atrial myopathy, gaps remain in understanding its underlying mechanisms and clinical implications. Advancing diagnostic tools, including imaging and biomarker discovery, will be crucial in identifying atrial myopathy at its earliest stages. Therapies targeting atrial myopathy itself, rather than its complications, hold promise for improving outcomes. Future research should focus on elucidating the molecular and cellular mechanisms driving age-related changes in the atria and exploring potential therapeutic targets.

In conclusion, the aging atrium presents both challenges and opportunities. By advancing our understanding of atrial myopathy and its relationship with aging, we can move toward a more comprehensive approach to cardiovascular disease prevention and management, ultimately improving the quality of life for our aging population.

## Figures and Tables

**Table 1.** Effects of aging on atrial structure, mechanical function, electrophysiological function, and metabolic function

Category	Age-related changes	Reference

Structure	Left atrial enlargement	Singh *et al*., 2022^[30]^
		Medrano *et al*., 2016^[31]^
		Pan *et al*., *Chest*, 2008^[32]^
	Increased atrial fibrosis	Lin *et al*., 2020^[35]^
		Gramley, 2009^[36]^
		Jansen *et al*., 2021^[37]^
	Decreased myocyte density	Takahashi *et al*., 2023^[38]^
	Increased myocyte hypertrophy	Clarke *et al*., 2017^[39]^
		Hayashi *et al*., 2002^[40]^
	Inflammatory cell infiltration	Wu *et al*., 2020^[41]^
		Tan *et al*., 2023^[42]^
Mechanical function	Decreased reservoir strain	Sugimoto *et al*., 2018^[46]^
		Nyberg *et al*., 2023^[47]^
		Singh *et al*., 2022^[30]^
		Zhang *et al*., 2023^[48]^
		Abou *et al*., 2017^[49]^
		Liao *et al*., 2017^[50]^
	Decreased conduit strain	Sugimoto *et al*., 2018^[46]^
		Nyberg *et al*., 2023^[47]^
		Singh *et al*., 2022^[30]^
		Zhang *et al*., 2023^[48]^
		Liao *et al*., 2017^[50]^
	Increased contractile strain	Singh *et al*., 2022^[30]^
		Liao *et al*., 2017^[50]^
		Boyd *et al*., 2011^[51]^
Electrophysiological Function	Abnormal P-wave terminal force in V1	Eranti *et al*., 2014^[53]^
		Wolder *et al*., 2023^[54]^
	Increased P-wave duration	Turhan *et al*., 2003^[55]^
		Jansen *et al*., 2017^[44]^
	Increased P-wave dispersion	Abou *et al*., 2017^[49]^
	Increased low voltage areas and conduction block	van der Does *et al*., 2021^[60]^
		Kistler *et al*., 2004^[61]^
Metabolic function	Impaired oxidative phosphorylation	Emelyanova *et al*., 2018^[65]^
	Impaired phosphotransfer and ATP cycling	Nemutlu *et al*., 2015^[66]^
	Altered fatty acid metabolism	Lee *et al*., 2019^[67]^

**Table 2.** Echocardiographic reference ranges for left atrial size and strain across different age groups

Source	^a^Sugimoto *et al*., 2018^[46]^			^b^Nyberg *et al*., 2023^[47]^					^c^Singh *et al*.,2022^[30]^		

Age range (years)	20–40	40–60	≥ 60	< 40	40–49	50–59	60–69	≥ 70	18–40	41–65	> 65
Left atrial maximal volume index (mL/m^2^) - males	-	-	-	-	-	-	-	-	16–41	16–46	18–48
Left atrial maximal volume index (mL/m^2^) - females	-	-	-	-	-	-	-	-	17–39	18–43	18–47
Reservoir strain (%) - overall	31	28	23	-	-	-	-	-	-	-	-
Reservoir strain (%) - males	-	-	-	24–53	21–53	20–49	16–46	13–40	25–63	23–61	24–57
Reservoir strain (%) - females	-	-	-	29–53	22–53	21–48	17–44	14–39	54–81	48–78	43–76
Conduit strain (%) - overall	16	12	12	-	-	-	-	-	-	-	-
Conduit strain (%) - males	-	-	-	13–36	8–32	7–27	3–23	1–19	18–50	12–43	10–36
Conduit strain (%) - females	-	-	-	15–37	10–34	9–28	4–23	2–19	19–52	12–42	9–36
Contractile strain (%) - overall	7	9	8	-	-	-	-	-	-	-	-
Contractile strain (%) - males	-	-	-	5–23	7–28	8–27	8–28	6–26	2–23	5–28	9–32
Contractile strain (%) - females	-	-	-	8–22	6–25	7–26	8–26	7–25	2–21	6–28	7–30

All strain measurements are provided as absolute values.

a Values represent the lower limit of normality;

b Values represent the range from (mean - 1.96 SD) to (mean + 1.96 SD);

c Values represent the range from lower to upper limit of normal.

**Table 3.** Markers and clinical manifestations of atrial myopathy

Markers of atrial myopathy	Reference

PTFV1	Chen *et al*., 2022^[52]^
Low-voltage areas	Sim *et al*., 2019^[62]^
	Yamaguchi *et al*., 2022^[64]^
Left atrial enlargement	Lisi *et al*., 2022^[106]^
	Patel *et al*., 2009^[107]^
Decreased left atrial strain	Lisi *et al*., 2022^[106]^
	Vieira *et al*., 2014^[105]^
	Kuppahally *et al*., 2010^[108]^
Late gadolinium enhancement	Siebermair *et al*., 2017^[120]^
	Oakes *et al*., 2009^[151]^
Mitral inflow velocity during late diastole	Thomas *et al*., 2020^[118]^
Mitral annular velocity during atrial contraction	Khankirawatana *et al*., 2004^[119]^
Clinical manifestations	
Atrial fibrillation	Goldberger *et al*., 2015^[6]^
	Rivner *et al*., 2020^[126]^
	Kallergis *et al*., 2014^[127]^
	Shen *et al*., 2019^[8]^
HFpEF	Omote *et al*., 2023^[7]^
	Melenovsky *et al*., 2015^[136]^
	Patel *et al*., 2021^[137]^
	Tamargo *et al*., 2020^[138]^
	Deedwania *et al*., 2010^[139]^
Thromboembolism	Goldberger *et al*., 2015^[6]^
	Shen *et al*., 2019^[8]^
	Smietana *et al*., 2019^[140]^
	Tan *et al*., 2023^[42]^

## Data Availability

Not applicable.
